# Sex Difference in Peripheral Inflammatory Biomarkers in Drug-Naïve Patients with Major Depression in Young Adulthood

**DOI:** 10.3390/biomedicines9070708

**Published:** 2021-06-22

**Authors:** Jinho Kim, Jong-Hoon Kim, Keun-A Chang

**Affiliations:** 1 Department of Health Sciences and Technology, GAIHST, Gachon University, Incheon 21936, Korea; jinho.k.0331@gmail.com (J.K.); jhnp@chol.com (J.-H.K.); 2 Department of Psychiatry, College of Medicine, Gachon University, Incheon 21565, Korea; 3 Gil Medical Center, Department of Psychiatry, Gachon University, Incheon 21565, Korea; 4 Neuroscience Research Institute, Gachon University, Incheon 21565, Korea; 5 Department of Pharmacology, College of Medicine, Gachon University, Incheon 21936, Korea

**Keywords:** major depression, young adult, inflammatory biomarkers, sex difference

## Abstract

The number of patients with major depressive disorder (MDD) is increasing worldwide. In particular, the early onset of MDD from adolescence to young adulthood is more problematic than the later onset. The specific and expeditious identification of MDD before the occurrence of severe symptoms is significant for future interventions or therapies; however, there is no accurate diagnostic marker that has sufficient sensitivity and specificity for clinical use. In the present study, to identify the possibility of blood markers for depression, we first measured the baseline inflammatory biomarkers in the peripheral blood of 50 treatment-naïve young adults with MDD and 50 matched healthy controls. We then analyzed the correlation between prospective biomarkers and depressive symptoms using scores from various clinical depression indices. We also identified differential responses between males and females in prospective biomarkers. In young adulthood, men with MDD had increased peripheral interleukin (IL)-17 levels, whereas women with MDD had significantly increased IL-1β, IL-6, and C-reactive protein (CRP) levels compared with healthy controls. However, tumor necrosis factor-α (TNF-α), CCL1, CCL2, adiponectin, and cortisol were not significantly different in young adult individuals with MDD. Higher levels of IL-17 in the male group and of IL-1β, IL-6, and CRP in the female group may have been associated with the clinical symptoms of MDD, including depressive moods, hopelessness, suicidal ideation, low self-esteem, and reduced psychological resilience. Our findings will be useful in developing diagnostic tools or treatments for MDD in young adulthood.

## 1. Introduction

Major depressive disorder (MDD), one of the common psychiatric diseases, is a very complex, multifactorial, and heterogeneous disorder [1]. MDD is the leading cause of disability worldwide and a major contributor to the global burden of disease [2,3]. The incidence of MDD markedly increases during early-stage and early-onset depression, and symptoms are often recurrent and persist into adulthood [4,5]. Moreover, early-onset depression occurs from the 20 s to early 30 s and peaks in young adulthood. Furthermore, in recent years, the increased rate of patients with MDD in young adulthood is higher than in other age groups [6,7]. Patients with early-onset depression have a poorer quality of life, experience more depressive episodes, and are more likely to exhibit social and occupational dysfunction and attempt suicide than those with late-onset MDD [8,9]. The occurrence and severity of MDD are higher among female patients than among male patients, and female patients have a 1.7-fold greater incidence than male patients [10]. However, the suicide rate of male patients at a young age is higher than that of female patients [11]. Therefore, the study of biomarkers for expeditious diagnosis and treatment, including age or sex factors, is extremely significant.

In biomarker studies of MDD, certain sociodemographic factors, such as age, sex, and obesity, have been associated with MDD [12]. However, previous studies have reported changes in peripheral biomarkers regardless of sex effect with participants of a wide age range (18–70 years) [13,14]. Factors such as age and related medications that may influence the development of biomarkers for MDD should be considered.

In addition, behavioral symptoms observed in patients with MDD, such as fatigue and pain, are associated with immune system activation [15]. Several pieces of biological evidence demonstrate that inflammatory cytokines play a significant role in the pathophysiology of MDD [16,17]. Hyperactivation or dysregulation of peripheral inflammatory cytokines also plays a pathophysiological role associated with the development of depression [18,19]. For example, individuals with MDD have elevated levels of inflammatory biomarkers, such as C-reactive protein (CRP), and pro-inflammatory cytokines, such as interleukin (IL)-6 and IL-1β, compared with healthy individuals [20]. A meta-analysis found that the chemokine levels of individuals with MDD are altered along with pro-inflammatory cytokines compared with those of healthy individuals [21]. The abnormal regulations of inflammation following obesity contribute more to the pathogenesis of MDD in women than in men [20,22]. These results suggest that women are more vulnerable to risk factors for depression than men.

Therefore, we evaluated the association between inflammatory biomarkers and depressive symptoms in young adult individuals with MDD, including through a comparative analysis between males and females. We then determined an appropriate biomarker for the diagnosis or treatment of MDD.

## 2. Materials and Methods

### 2.1. Participants

The study protocol (GFIRB2018-156: 15 May 2018) was approved by the Institutional Review Board of the Gachon University Gil Medical Center, and all procedures used in the study were conducted as per international ethical standards and the Declaration of Helsinki. Written informed consent was obtained from all participants after they had received a full explanation of the study procedures. Our study focused on measuring inflammatory cytokine and chemokine levels in a homogeneous group of treatment-naïve young adults with MDD. As such, the first inclusion criterion was age from 19 to 35 years. Other inclusion criteria were as follows: diagnosis of MDD based on the Diagnostic and Statistical Manual of Mental Disorders, Fourth Edition (DSM-IV) [23], which was established by the Structured Clinical Interview for DSM-IV (SCID-IV) [24] with no other current Axis I diagnosis, and no past or current substance abuse/dependence, no history of medical or neurological disorders, no past or current history of psychiatric treatments, and no past or current use of any psychotropic medications (e.g., antidepressants, benzodiazepines/anxiolytics, hypnotics, antipsychotics, or mood stabilizers). Patients with comorbid anxiety disorders, such as generalized anxiety disorder, panic disorder, phobic disorders, obsessive—compulsive disorder, or posttraumatic stress disorder, were excluded. A total of 50 patients who met the inclusion criteria were enrolled (Table 1). To compare the findings of the patient group, 50 matched healthy controls (HCs) who met the criteria of no past or current psychiatric, neurological, or medical disorders and no past or current use of medications known to affect the central nervous system were recruited, provided written informed consent, and underwent the same protocols. Patients with MDD were recruited from outpatient clinics and through local advertisements, and control subjects were recruited through local advertisements.

### 2.2. Clinical Data

Clinical assessments were conducted using the Beck Depression Inventory (BDI), State-Trait Anxiety Inventory-X-1 (STAI-X-1), Hamilton Rating Scale for Depression-17 (HAMD-17), Rosenberg Self-Esteem (RSE) Scale, Barratt Impulsiveness Scale (BIS), Beck Hopelessness Scale (BHS), KAIST Scale for Suicidal Ideation (KSI), and Resilience Appraisal Scale (RAS).

BDI: The BDI is a 21 item self-rating inventory that measures the presence and degree of depressive symptoms and is one of the most widely used scales for assessing the subjective severity of depression. The items include symptoms and characteristics commonly found in depressed individuals, such as depressive mood, sadness, guilt feeling, self-dislike, sleep difficulty, and social withdrawal. Each item is scored on a four-point rating scale [25].

STAI-X-1: The STAI-X-1 is a 20 item self-rating scale that assesses the levels of anxiety [26]. This questionnaire measures the current state of anxiety and related symptoms characterized by subjective feelings of tension, apprehension, agitation, and heightened autonomic nervous system symptoms. Each item is scored on a four-point rating scale.

HAMD-17: The HAMD is the most widely used clinician-administered depression assessment scale [27]. The scale measures core symptoms of depression, including depressed mood, loss of interest, feeling of guilt, psychomotor retardation, insomnia (early, middle, and late), weight change, suicidal ideation, and impairment of functioning. The total score can range from 0 to 52.

RSE Scale: The RSE scale is a widely used self-rating scale with 10 items, evaluating an individual’s self-esteem [28]. The RSE assesses general self-worth by measuring both positive and negative feelings about self. Each item is scored on a four-point scale.

BIS: The BIS is the most widely administered self-report inventory for the assessment of impulsiveness [29]. It is a 30 item self-administered questionnaire that assesses the control of thoughts and behavior. Each item is scored on a four-point scale. The BIS measures comprehensive aspects of impulsivity, including non-planning impulsiveness, motor impulsiveness, and attentional impulsiveness.

BHS: The BHS is a 20 item self-rating scale used to assess the aspects of hopelessness characterized by a generalized negative future expectation [30]. All items are scored on a true–false rating scale. The BHS measures the major aspects of hopelessness, such as pessimistic thoughts, loss of motivation, expectations of failure, and negative attitudes about the future.

KSI: The KSI measures various levels of suicidal ideation over the previous 2 weeks or the last year on a scale ranging from mild (“I would rather fall asleep and not wake up”) to severe (“I will carry out my thoughts of wanting to make my own life”) [31]. The KSI is composed of 14 items, and each item is scored on a four-point scale.

RAS: The RAS is a commonly used rating scale to measure psychological resilience and positive self-appraisals [32]. The RAS measures the appraisal of the individual’s ability to cope with emotions, solve problems, and gain social support. These types of positive self-appraisals may be particularly significant in buffering individuals from suicidal thoughts in the face of stressful life events [32]. The RAS is a 12 item self-rating instrument, and each item is scored on a five-point scale.

For the BDI, STAI-X-1, HAMD-17, BIS, BHS, and KSI, higher scores indicate more severe symptoms, whereas higher RSE and RAS scores indicate higher self-esteem and higher resilience related to social support, emotional regulation ability, and problem-solving.

### 2.3. Serum and Plasma Separation

Blood (10 mL) was collected from each participant from the forearm vein into a Cell Preparation Tube (CPT) (BD, Franklin Lakes, NJ, USA) and Serum Separator Tube (SST) (BD) under strict aseptic conditions. Samples for the serum were kept at room temperature for 30–40 min to allow clot formation and then centrifuged for 20 min at 1600× *g* at room temperature. To separate the serum, the SST was centrifuged for 10 min at 3000 rpm at 4 °C. After separating the serum and plasma, a protease inhibitor cocktail (EMD Biosciences, Inc., Darmstadt, Germany) and phosphatase inhibitor cocktail (Sigma-Aldrich, Inc., St. Louis, MO, USA) were added to the samples. Serum and plasma samples were aliquoted and immediately stored at −80 °C until further analysis. Aliquots were thawed on the day of the analysis.

### 2.4. Measurement of Biomarkers

The levels of inflammatory cytokines and chemokines were established in plasma samples and the levels of cortisol and adiponectin were established in serum samples. Undiluted samples were used for inflammatory cytokines and chemokines, and samples for CRP and adiponectin were diluted 200-fold. All biomarkers were analyzed in duplicate using the following enzyme-linked immunosorbent assay (ELISA) kits: IL-1β (KET6013; Abbkine, Wuhan, China), IL-6 (KET6017; Abbkine, Wuhan, China), TNF-α (ADI-900-099; Enzo, Madison Avenue, New York, NY, USA), IL-17 (KET6022; Abbkine, Wuhan, China), CRP (DCRP00; R&D Systems, Minneapolis, MN, USA), adiponectin (DRP300; R&D Systems, Minneapolis, MN, USA), cortisol (ADI-900-071; Enzo, New York, NY, USA), CCL1 (MBS824930; MyBioSource, San Diego, CA, USA), and CCL2 (LS-F146; LSBio, Seattle, WA, USA), according to the manufacturer’s instructions. The standard solutions were respectively prepared using the reagents provided in each kit. Plates were read at 450 nm using a VICTOR X4 Multimode Plate Reader (PerkinElmer, Waltham, MA, USA). Protein quantification was performed using the mean value of the duplicate samples.

### 2.5. Statistical Analysis

All data are presented as means ± standard deviation. Data were analyzed using a two-tailed *t*-test between the two groups. A two-way analysis of variance followed by Tukey’s multiple comparison tests were used to compare the two groups of sex. A *p*-value of <0.05 was considered statistically significant for all tests. Correlations were assessed using the nonparametric Spearman’s rank correlation test. The heatmap shows the correlation between inflammatory biomarkers and variables. The tables show regression lines with 95% confidence intervals and the *r*- and *p*-values after the correlation test. ROC analyses were conducted under the nonparametric distribution assumption for the standard error of area to determine the capacities of the measurements to discriminate depression in young adulthood from the level of biomarkers. The cutoff value was determined using Youden’s J statistic. All statistical analyses were performed using GraphPad Prism 8.4.2 (679) software (GraphPad Software Inc., San Diego, CA, USA).

## 3. Results

This study included 50 participants with MDD and 50 HCs in young adulthood. MDD and HC participants were selected in equal proportions for men and women. Table 1 summarizes the clinical and demographic characteristics of the study. No significant differences in demographic or clinical variables were identified between patients with MDD and HCs.

### 3.1. Profiles of Baseline Inflammatory Cytokines in Young Adult Patients with MDD

**Figure 1:**
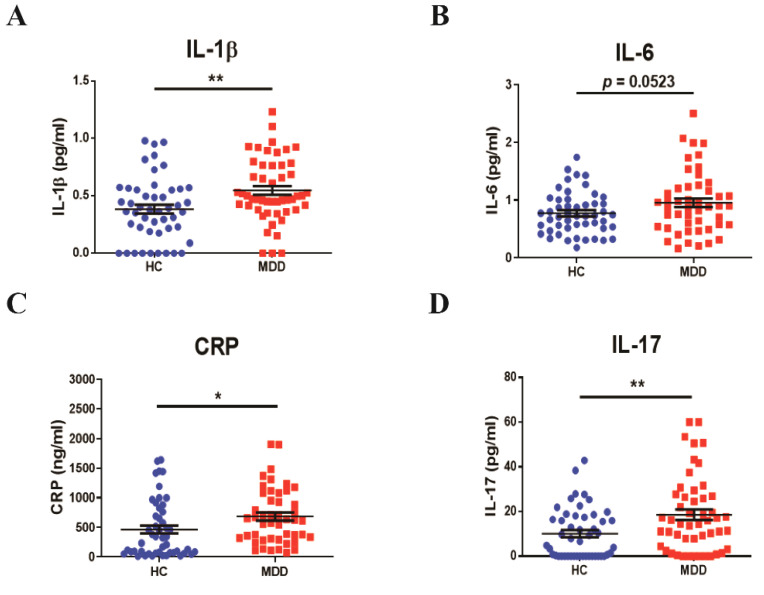
The baselines of the inflammatory cytokines in young adult patients with major depression and in the age-matched controls. The concentrations of (**A**) IL-1β, (**B**) IL-6, (**C**) CRP, and (**D**) IL-17 in serum were quantified using the ELISA system. The serum was separated from peripheral blood in participants. *n* = 50 for each group. * *p* < 0.05 and ** *p* < 0.01 by two-tailed *t*-test. Data are presented as means ± SD.

To observe the baseline peripheral blood biomarkers in young adult individuals in the MDD or age-matched normal healthy (HC) groups, we first measured the levels of inflammatory-mediated markers (IL-1β, IL-6, CRP, IL-17, TNF-α, CCL1, CCL2, and adiponectin) and cortisol using the ELISA system. The IL-1β level (MDD, 0.5478 ± 0.2770 pg/mL, vs. HC, *p* < 0.01, Figure 1A) was significantly higher in the young adult MDD group than in the HC group. In the peripheral blood of the MDD group, the IL-6 level (MDD, 0.9545 ± 0.5388 pg/mL, vs. HC, *p* = 0.0523, Figure 1B) also increased, but it was not significant. In addition, the peripheral CRP (MDD, 683.1 ± 465.8 ng/mL, vs. HC, *p* = 0.0269, Figure 1C) and IL-17 (MDD, 18.58 ± 16.75 pg/mL, vs. HC, *p* = 0.0042, Figure 1D) levels of the MDD group significantly increased compared with those of the HC group. However, there were no significant differences in TNF-α, CCL1, CCL2, cortisol, and adiponectin between the groups (Table S1).

### 3.2. Sex Differences in Inflammatory Response in Young Adult Patients with MDD

**Figure 2:**
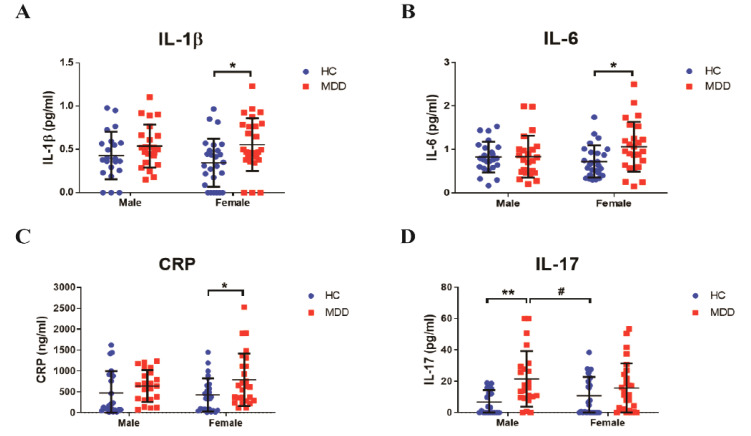
Different tendencies of inflammatory biomarkers between males and females. (**A**) IL-1β, (**B**) IL-6, (**C**) CRP, and (**D**) IL-17 were analyzed using the serum of participants. *n* = 23–27 for each group. * *p* < 0.05 and ** *p* < 0.01 vs. control male group, # *p* < 0.05 vs. control female group by two-way ANOVA test. Data are presented as means ± SD.

After confirming the baseline inflammatory cytokines between the groups, we separately analyzed the levels of inflammatory biomarkers by sex. In female subjects with MDD, the levels of IL-1β (MDD, 0.5555 ± 0.3033 pg/mL, vs. female HC, 0.3455 ± 0.2777 pg/mL, *p* = 0.0355, Figure 2A), IL-6 (MDD, 1.064 ± 0.5719 pg/mL, vs. female HC, 0.7237 ± 0.3694 pg/mL, *p* = 0.0355, Figure 2B), and CRP (MDD, 790.8 ± 632.4 ng/mL, vs. female HC, 415.2 ± 361.5 ng/mL, *p* = 0.0497, Figure 2C) significantly increased in the serum compared with those in the female HC group, but not in the male group. However, the IL-17 levels (MDD, 21.55 ± 17.70 pg/mL, vs. male HC, 6.755 ± 7.536 pg/mL, *p* = 0.0039; vs. female HC, *p* = 0.0416; Figure 2D) in the serum significantly increased in young adult male patients with MDD compared with those in the male and female HC groups. Nevertheless, there were no statistically significant differences in the inflammatory biomarkers between male and female patients.

### 3.3. Correlation between Depressive Symptoms and Inflammatory Biomarkers in Young Adulthood

After determining the baseline inflammatory biomarkers, we analyzed the association between clinical severity (BDI, STAI-X-1, HAMD-17, BIS, RSE, BHS, KSI, and RAS) and the levels of inflammatory biomarkers in the peripheral blood of HC and patients with MDD (Table S2). In the young adult MDD group, IL-1β in the serum was positively correlated with the BDI (*p* = 0.0007), STAI-X-1 (*p* < 0.0001), HAMD-17 (*p* = 0.0111), and BHS (*p* < 0.0001) scores but negatively correlated with the RSE (*p* = 0.0005) and RAS (*p* = 0.0046) scores. IL-6 was positively correlated with the STAI-X-1 (*p* = 0.0162), BHS (*p* = 0.0159), and KSI (*p* = 0.0133) scores but negatively correlated with the RSE (*p* = 0.0334) and RAS (*p* = 0.0176) scores. The CRP level was positively correlated only with the HAMD-17 (*p* = 0.0432) score. In addition, the IL-17 level was positively correlated with the BDI (*p* = 0.0189) and HAMD-17 (*p* = 0.0282) scores but negatively correlated with the RAS (*p* = 0.2711) score.

**Figure 3:**
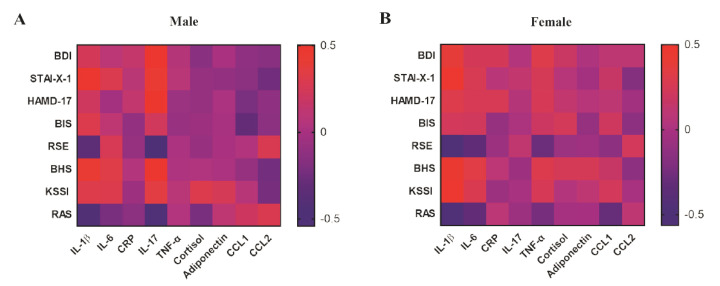
The heatmap indicates the *r*-value of the correlation between the clinical scores and inflammatory biomarkers of participants in young adulthood. The red color means a positive correlation, and the blue color means a negative correlation in both male and female participants. (**A**) Male (HC, *n* = 24; MDD, *n* = 23) and (**B**) female (HC, *n* = 26; MDD, *n* = 27).

We also analyzed sex-related correlations between inflammatory biomarkers and clinical scores (Figure 3 and Table S2). In the male group (Figure 3A and Table S3), the IL-1β level was positively correlated with the STAI-X-1 (*p* = 0.0062) and BHS (*p* = 0.0085) scores but negatively correlated with the RSE (*p* = 0.0404) and RAS (*p* = 0.0173) scores. The IL-6 level in male serum was positively correlated only with the BHS (*p* = 0.0283) score. However, there was no significant correlation between the CRP level and scores of and clinical symptoms in the male group. IL-17 in male serum especially was positively correlated with the BDI (*p* = 0.0005), HAMD-17 (*p* = 0.0008), BHS (*p* = 0.0020), and KSI (*p* = 0.0487) scores but negatively correlated with the RSE (*p* = 0.0058) and RAS (*p* = 0.0014) scores.

Unlike the correlation in the male group, the female group had a significant correlation between the scores of depressive and clinical symptoms and IL-1β and IL-6 levels (Figure 3B and Table S4). The IL-1β level in female serum was positively correlated with the BDI (*p* = 0.0056), STAI-X-1 (*p* = 0.0019), HAMD-17 (*p* = 0.0286), BHS (*p* = 0.0012), and KSI (*p* = 0.0018) scores but negatively correlated with the RSE (*p* < 0.0001) and RAS (*p* = 0.0002) scores. The IL-6 level in female serum was positively correlated with the BHS (*p* = 0.0283) score but negatively correlated with the RSE (*p* = 0.0297) and RAS (*p* = 0.0409) scores.

### 3.4. Predictable Diagnostic Marker from the Baseline of the Inflammatory Biomarkers in Young Adult Patients with MDD

**Figure 4:**
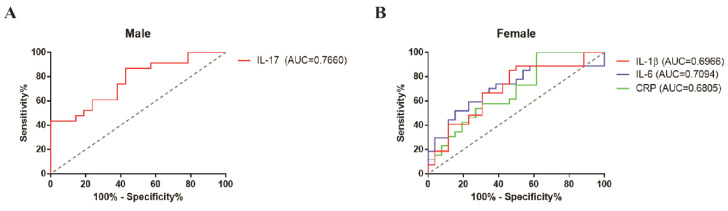
Receiver operating characteristic (ROC) analyses of IL-17 in the male group (**A**) and IL-1β, IL-6, and CRP in the female group (**B**), indicating moderate diagnostic accuracy of each elevated inflammatory biomarker. AUC, area under the curve. Male (HC, *n* = 24; MDD, *n* = 23) and female (HC, *n* = 26; MDD, *n* = 27). The cutoff value was determined using Youden’s J statistic.

After confirming the association between the inflammatory biomarkers and clinical scores, the ROC curve in males and females was analyzed to estimate the predictive potential and accuracy in inflammatory biomarkers of young adult patients with MDD. We found 86.96% sensitivity and 57.14% specificity in the IL-17 level (area under the curve (AUC) = 0.7660, *p =* 0.002546) in the male group (Figure 4A). Based on Youden’s J statistic, the optimal cutoff value of IL-17 in the male group was determined to be 5.826 pg/mL. In the female group (Figure 4B), we also found 85% sensitivity and 53.35% specificity in the IL-1β level (AUC = 0.6966, *p =* 0.01411); 51.85% sensitivity and 84.62% specificity in the IL-6 level (AUC = 0.7094, *p =* 0.008938); and 100% sensitivity and 38.46% specificity in the CRP level (AUC = 0.6805, *p =* 0.02561). In the female group, the best cutoff values of IL-1β, IL-6, and CRP were 0.3562 pg/mL, 1.039 pg/mL, and 105.4 ng/mL, respectively. However, ROC curves of IL-1β, IL-6, and CRP in the male group and IL-17 in the female group showed no significant differences between the HC and MDD groups (Figure S1).

## 4. Discussion

This study aimed to observe and discover the inflammatory biomarkers in patients with MDD in young adulthood. Recently, because the number of patients with MDD in young adulthood is increasing, several studies have been performed to prove the pathophysiology of depression; however, the specific marker remains elusive. Studies on biomarker development are important in the diagnosis or treatment of MDD from various perspectives, such as regarding the brain-derived neurotrophic factor, oxidative stress, and the hypothalamic–pituitary–adrenal axis [5,33,34]. Here, based on inflammation, we investigated peripheral changes to find prospective evidence for clinical biomarkers in MDD.

Most studies have focused on a wide age range (18–65 years) or adolescent patients with MDD; studies on young adults with MDD have been insufficient. The onset of MDD most frequently occurs in adolescence or young adulthood, and these patients experience lifetime MDD episodes [7,35]. In a longitudinal prospective study from childhood to adulthood, up to 72% of those who recovered from the first episode of MDD had recurrent MDD with intervals 3–5 years [36]. In addition, chronic episodes are associated with a higher level of depressive symptoms and mental dysfunction as well as impairment of several psychosocial domains [37,38]. Considering the need for research on young adults with MDD, we focused on college-age adults (average age 24 years) with MDD.

Based on previous studies showing that an upregulated inflammatory response was associated with depressed mood [39], we also observed the inflammatory biomarkers in the peripheral blood of young adult patients with MDD and age-matched controls. Consequently, we identified that IL-1β (*p* < 0.01), IL-6 (*p* = 0.0523), CRP (*p* < 0.05), and IL-17 (*p* < 0.01) were upregulated in the MDD group. However, there were no significant differences in the baseline or association of peripheral TNF-α, CCL1, CCL2, adiponectin, and cortisol levels in young adult patients with MDD compared with controls. The increase in TNF-α level was observed more in elderly patients than in younger-aged patients with MDD [40]. Furthermore, it has been reported that, compared with controls, depressed patients showed higher levels of CCL2 in the serum but not in plasma [21]. In the case of adiponectin, which is an anti-inflammatory substance, different races showed different trends [41]. Regarding cortisol, the results of this study had some limitations. It has been reported that both patients with depression and normal individuals showed higher cortisol levels and significant differences in the morning, and the difference between the groups decreased in the afternoon [42]. A recent meta-analysis of 20 studies also reported that the small observed difference in morning and evening cortisol levels did not reliably distinguish depressed and nondepressed individuals, except for those more severely depressed [43]. Therefore, sampling time should be considered to demonstrate the potential of a biomarker of cortisol in young adults. However, the inconsistent sampling times in our study were a limitation, which appears to have affected the results for cortisol.

Interestingly, we identified different responses of inflammatory biomarkers according to sex. Peripheral IL-17 was significantly upregulated in the male group with MDD. IL-17, which is produced by several innate/adaptive immune cells, induces several inflammatory mediators. In clinical studies, higher IL-17 levels were associated with anhedonia severity in men, but not in women [44,45]. Our results are also consistent with previous reports that the increase in IL-17 level, including Th17 cells, was associated with depression-like behavior in rodent models [46,47]. Conversely, serum IL-1β, IL-6, and CRP levels significantly increased only in the female group with MDD compared with those in the age- and sex-matched controls. It is considered that stress resilience or inflammatory response is caused by sex differences [22,48,49]. Specifically, sex differences within the immune system may contribute to the amplified risk of MDD in women [50]. In addition, fluctuations in ovarian hormone affect susceptibility to stress as well as brain structure and function, including inflammatory response.

It is well-known that women are much more likely to suffer from stress-related mental disorders than men [51]. Several studies have found that not only are women twice as likely to experience depression as men, but they are also significantly more vulnerable after exposure to an inescapable stressful event [51]. Numerous studies have argued that selective serotonin reuptake inhibitor (SSRI) antidepressant therapy is more effective in female patients than in male patients [52]. In the preclinical study, the treatment of fluoxetine, which is one of the SSRI antidepressants, acted as a different response between male and female mice [53,54]. Chronic treatment with fluoxetine for more than two weeks prevented the learning deficit in females exposed to stress but did not alter the males’ responses to stress [53]. Hodes et al. demonstrated that pharmacokinetic differences in fluoxetine metabolism may contribute to sex differences in the effect of fluoxetine on neurogenesis, leading to greater responsiveness to fluoxetine in females than in males with greater brain plasticity [54].

Depressive symptoms, such as depressed mood, loss of interest, and anxiety, are associated with the major depressive episode and have been known to differ between men and women [55,56]. In the correlation between baseline inflammatory biomarkers and clinical scores, we found that increased IL-17 levels in the male group were strongly correlated with self-report inventory or indication of depression of mood, feeling of guilt, anhedonia, and suicide ideation using the BDI, HAMD-17, BHS, and KSI scores. However, the RSE scale and RAS were negatively correlated with IL-17 concentration in the male group. In the female group, IL-1β had a positive correlation with the BDI, STAI-X-1, HAMD-17, BHS, and KSI scores and a negative correlation with the RSE and RAS scores in young adulthood. Although several studies have reported that several inflammatory cytokines were correlated with depressive symptom scores [57,58], some studies have not [59,60]. As depression has diverse clinical symptoms, we used various clinical assessments, including depressive moods, hopelessness, anxiety, and suicide ideation, as well as self-esteem or resilience, to improve the accuracy of the association. To prove the association between biomarkers and depressive symptoms in young adulthood, we suggest that IL-17 and IL-1β are significant diagnostic targets in male and female patients, respectively.

Our investigation has several limitations. First, large samples and targets for the verification of inflammatory biomarkers for patients with MDD in young adulthood would be helpful. Although the number of samples in our study was adequate to support the peripheral changes in young adult patients, a larger sample would further support our opinion. Second, owing to insufficient sample quantity for the ELISA system, we analyzed chemokines such as CCL1 and CCL2 in plasma levels. Third, we need to prove the recovery of abnormal regulation of inflammatory biomarkers in young adulthood using antidepressants, including interaction effects with sex. Fourth, we need to check the menstrual cycle in the female group because it is closely related to psychosocial stress. Finally, further studies on the causes of sex differences in inflammatory biomarkers, such as hormones, are warranted.

## 5. Conclusions

This study showed that the baseline levels of peripheral cytokines, including IL-17, IL-1β, IL-6, and CRP, increased in young adult patients with MDD. Moreover, higher levels of IL-17 in the male group and those of IL-1β, IL-6, and CRP in the female group may have been associated with the clinical symptoms of MDD, including depressive moods, hopelessness, suicidal ideation, low self-esteem, and reduced psychological resilience. This study supports the hypothesis that these inflammatory biomarkers may help in the development of diagnostic or therapeutic targets in young adult patients with MDD.

## Figures and Tables

**Table 1:** Baseline demographic and clinical data.

	Total		Male		Female	
	HC	MDD	HC	MDD	HC	MDD
Demographic information						
Number (*n*)	50	50	24	23	26	27
Age (SD, year)	24.80 ± 3.22	24.18 ± 3.74	24.17 ± 2.24	24.52 ± 2.86	25.52 ± 3.86	24.19 ± 3.69
BMI (SD)	21.83 ± 4.77	22.68 ± 4.04	22.60 ± 4.87	24.03 ± 3.78	20.89 ± 5.01	23.01 ± 4.28
Smoking (%)	7 (18%)	17 (34%)	7 (26%)	12 (52%)	0 (0%)	5 (19%)
Non-smoking (%)	43 (82%)	33 (66%)	17 (74%)	11 (48%)	26 (100%)	22 (81%)
Consumption of Alcohol (%, three times a week)	2 (4%)	8 (16%)	1 (4%)	4 (17%)	1 (4%)	4 (15%)
Non-frequent consumption (%, under two times a week)	48 (96%)	42 (84%)	23 (96%)	20 (83%)	25 (96%)	23 (85%)
Education (SD, years)	15.60 ± 1.61	14.90 ± 1.55	15.67 ± 2.04	14.83 ± 1.83	15.62 ± 1.19	15.00 ± 1.31
Clinical information						
Age of onset (SD, year)	NA	19.76 ± 5.67	NA	20 ± 4.12	NA	18.91 ± 6.09
Number of episodes (SD)	NA	2.43 ± 1.08	NA	2.39 ± 1.09	NA	2.48 ± 1.12
BDI	1.54 ± 1.64	26.44 ± 8.91 ****	55.35 ± 1.51	26.84 ± 1.29 ****	1.81 ± 1.74	25.85 ± 9.57 ****
STAI-X-1	31.73 ± 5.91	56.84 ± 8.87 ****	31.73 ± 5.79	57.24 ± 1.38 ****	31.74 ± 6.18	56.09 ± 8.77 ****
HAMD-17	NA	23.32 ± 6.94	NA	23.16 ± 1.04	NA	23.22 ± 8.35
BIS	55.40 ± 9.60	70.68 ± 12.04 ****	55.35 ± 7.48	69.20 ± 10.34 ****	55.44 ± 11.17	71.78 ± 13.24 ****
RSE	34.16 ± 4.20	20.00 ± 5.47 ****	34.94 ± 4.01	19.40 ± 6.21 ****	33.42 ± 4.35	20.44 ± 4.93 ****
BHS	1.16 ± 1.54	12.53 ±5.54 ****	1.17 ± 1.58	13.75 ± 5.34 ****	1.16 ± 1.54	11.63 ± 5.60 ****
KSI	0.57 ± 2.06	32.41 ±25.03 ****	0.67 ± 2.83	35.33 ± 28.79 ****	0.47 ± 0.96	30.41 ± 22.61 ****
RAS	49.65 ± 5.71	32.62 ± 7.54 ****	50.61 ± 4.98	33.93 ± 7.30 ****	48.74 ± 3.62	31.73 ± 7.73 ****

BDI, Beck Depression Inventory; STAI-X-1, State-Trait Anxiety Inventory-X-1; HAMD-17, Hamilton Rating Scale for Depression-17; BIS, Barratt Impulsiveness Scale; RSE, Rosenberg Self-Esteem Scale; BHS, Beck Hopelessness Scale; KSI, KAIST Scale for Suicidal Ideation; RAS, Resilience Appraisals Scale. NA, Not Applicable. **** *p* < 0.01 compared with the HC subjects, using two-tailed *t*-test.

## Data Availability

The datasets used and/or analyzed during the current study are available from the corresponding author on reasonable request.

## Supplementary Materials

The following are available online at https://www.mdpi.com/article/10.3390/biomedicines9070708/s1, Table S1: Results of baselines for inflammatory cytokines, Table S2: Correlation of depression index scores and inflammatory biomarkers, Table S3: Correlation of depressive scores and inflammatory biomarkers in the male group, Table S4: Correlation of depressive scores and inflammatory biomarkers in the female group, Figure S1: The results of receiver operating characteristic (ROC) analysis for inflammatory biomarkers in young adulthood.

## Abbreviations

BDI	Beck Depression Inventory
BHS	Beck Hopelessness Scale
BIS	Barratt Impulsiveness Scale
HAMD-17	Hamilton Rating Scale for Depression-17
HC	healthy control
KSI	KAIST Scale for Suicidal Ideation
MDD	major depressive disorder
RAS	Resilience Appraisal Scale
RSE	Rosenberg Self-Esteem Scale
STAI-X-1	State-Trait Anxiety Inventory-X-1
SSRI	Serotonin Selective Reuptake Inhibitor
