# Supplementary material for: Sex Difference in Peripheral Inflammatory Biomarkers in Drug-Naïve Patients with Major Depression in Young Adulthood
Source: Biomedicines. 2021 Jun 22;9(7):708. doi: 10.3390/biomedicines9070708 (PMC8301344; doi:10.3390/biomedicines9070708)
Supplement: Supplementary file 1 [file biomedicines-09-00708-s001.zip › biomedicines-1245099-supplementary.pdf]

Sex difference in peripheral inflammatory biomarkers in drug-naïve patients with major depression in young adulthood

Jinho Kim[1], **Jong-hoon Kim**[1,2,3,4], **and Keun-A Chang**[1,4,5,*]

[1]Department of Health Sciences and Technology, GAIHST, Gachon University, Incheon 21936, Korea

[2] Department of Psychiatry, College of Medicine, Gachon University, Incheon 21565, Korea

[3] Department of Psychiatry, Gil Medical Center, Gachon University, Incheon 21565, Korea

[4] Department of Pharmacology, College of Medicine, Gachon University, Incheon 21936, Korea

[5] Neuroscience Research Institute, Gachon University, Incheon 21565, Korea

* Correspondence to: Keun-A Chang, PhD, Department of Pharmacology, College of Medicine, Gachon University, Incheon 21936, Korea; keuna705@gachon.ac.kr

Table S1. Results of baseline in inflammatory biomarkers

Biomarkers	Total population				Male				Female			
	Control		MDD		Control		MDD		Control		MDD	
	Mean	SD	Mean	SD	Mean	SD	Mean	SD	Mean	SD	Mean	SD
**TNF-α (pg/ml)**	9.868	6.384	9.316	5.295	24.36	25.86	10.38	5.798	8.327	5.794	26.25	36.96
**Cortisol (ng/ml)**	43.06	10.92	42.68	12.20	45.54	10.27	43.53	13.43	39.93	11.30	42.03	11.64
**Adiponectin (ng/ml)**	8026	3999	7692	4020	7194	3998	7024	3923	8795	3920	8261	4087
**CCL1 (pg/ml)**	467.1	510.4	381.5	475.5	471.1	459.2	262.6	386.3	240.6	402.2	289.9	413.3
**CCL2 (pg/ml)**	25.98	20.94	23.02	28.05	31.61	22.80	27.68	32.11	15.30	8.335	10.30	7.066

Table S2. Correlation of depression index scores and inflammatory biomarkers

Inflammatory biomarkers		Indexes of depressive symptoms							
		BDI	STAI-X-1	HAMD-17	BIS	RSE	BHS	KSSI	RAS
Il-1β	r	**0.3376**	**0.5005**	**0.2556**	0.1309	**-0.3755**	**0.4497**	0.2275	**-0.3303**
	p	**0.0007*** **	**<0.0001******	**0.0111***	0.2189	**0.0005*****	**<0.0001******	0.0547	**0.0046****
	95% CI	0.1492, 0.5024	0.3041, 0.6561	0.06013, 0.4321	-0.07841, 0.3291	-0.5479, -0.1725	0.1222, 0.5109	-0.004495, 0.4362	-0.5221, -0.1068
IL-6	r	0.1681	**0.2805**	0.1407	0.1872	**-0.2352**	**0.2656**	**0.2884**	**-0.2772**
	p	0.0981	**0.0162***	0.1669	0.0773	**0.0334***	**0.0159***	**0.0133***	**0.0176***
	95% CI	-0.03144, 0.3547	0.05383, 0.4796	-0.05941, 0.3300	-0.02078, 0.3796	-0.4303, -0.01914	0.05154, 0.4564	0.06246, 0.4863	-0.4769, -0.05023
CRP	r	0.1565	0.02614	**0.2068**	-0.06233	-0.04811	0.02740	-0.08822	0.04820
	p	0.1279	0.8299	**0.0432***	0.5640	0.6717	0.8093	0.4677	0.6920
	95% CI	-0.04550, 0.3461	-0.2102, 0.2596	0.006558, 0.3911	-0.2683, 0.1491	-0.2651, 0.1735	-0.1935, 0.2457	-0.3167, 0.1499	-0.1890, 0.2801
IL-17	r	**0.2368**	0.1728	**0.2218**	0.1777	0.1566	0.2073	0.1727	**-0.2711**
	p	**0.0189***	0.1438	**0.0282***	0.1298	0.1574	0.0600	0.1441	**0.0204***
	95% CI	0.04025, 0.4158	-0.05970, 0.3875	0.02441, 0.4026	-0.3602, 0.06119	-0.3602, 0.06119	-0.008824, 0.4049	-0.05982, 0.3874	-0.4718, -0.04368
TNF-α	r	0.1128	0.07512	-0.1592	0.1095	0.02227	-0.07740	-0.1395	-0.03842
	p	0.3386	0.5821	0.1611	0.3041	0.8569	0.5304	0.2922	0.7727
	95% CI	-0.1188, 0.3328	-0.1916, 0.3315	-0.3674, 0.06423	-0.05297, 0.3904	-0.2174, 0.2594	-0.3102, 0.1641	-0.3820, 0.1210	-0.2917, 0.2199
Cortisol	r	0.01793	-0.1866	0.02334	0.1093	-0.06521	0.1426	0.02642	0.06072
	p	0.8594	0.1277	0.8177	0.2997	0.5556	0.1957	0.8232	0.6073
	95% CI	-0.1792, 0.2136	-0.2498, 0.2071	-0.1739, 0.2188	-0.09777, 0.3073	-0.2758, 0.1514	-0.07412 0.3464	-0.2034, 0.2534	-0.1702, 0.2853
Adiponectin	r	0.01455	-0.04795	0.03657	-0.05070	-0.03754	0.03394	0.1627	-0.009076
	p	0.8857	0.6850	0.7179	0.6313	0.7346	0.7592	0.1661	0.9388
	95% CI	-0.1824, 0.2104	-0.2735, 0.1826	-0.1611, 0.2314	-0.2529, 0.1558	-0.2500, 0.1783	-0.1818, 0.2466	-0.06843, 0.3772	-0.2371, 0.2199
CCL1	r	-0.003289	0.04097	-0.03231	0.01168	-0.04715	0.05294	0.1755	-0.05959
	p	0.9741	0.7289	0.7496	0.9120	0.6702	0.6324	0.1347	0.6140

	**95% CI**	-0.1996, 0.1933	-0.1894, 0.2670	-0.2273, 0.1652	-0.1937, 0.2160	-0.2590, 0.1690	-0.1634, 0.2644	-0.05524, 0.3885	-0.2843, 0.1713
**CCL2**	**r**	-0.03186	-0.1866	-0.01424	-0.1595	**0.2303**	-0.1429	-0.09173	0.1873
	p	0.7630	0.1277	0.8928	0.1448	**0.0423***	0.2119	0.4569	0.1262
	**95% CI**	-0.2352, 0.1741	-0.4069, 0.05434	-0.2185, 0.1912	-0.3604, 0.05557	0.008085, 0.4308	-0.3543, 0.08226	-0.3231, 0.1500	-0.05360, 0.4076

Table S3. Correlation of depressive scores and inflammatory biomarkers in male group

Inflammatory biomarkers		Indexes of depressive symptoms							
		BDI	STAI-X-1	HAMD-17	BIS	RSE	BHS	KSSI	RAS
IL-1β	r	0.2444	0.4809	0.2125	0.3024	-0.3432	0.4318	0.3124	-0.4247
	p	0.1057	**0.0062****	0.1611	0.0650	**0.0404***	**0.0085****	0.0870	**0.0173***
	95% CI	-0.05306, 0.5019	0.1525, 0.7137	-0.08650, 0.4764	-0.01926, 0.5673	-0.6038, -0.01649	0.1203, 0.6659	-0.04721, 0.6004	-0.6772, -0.08269
IL-6	r	0.09509	0.2964	-0.01284	0.1217	0.2685	0.3270	0.3019	-0.2167
	p	0.5249	0.0940	0.9317	0.4545	0.1032	**0.0283***	0.0877	0.2259
	95% CI	-0.1975, 0.3722	-0.05231, 0.5807	-0.2990, 0.2754	-0.1974 0.4174	-0.05612, 0.5417	0.03694, 0.5662	-0.04629, 0.5847	-0.5213, 0.1369
CRP	r	0.1545	0.09208	0.1410	-0.1058	-0.08821	0.05216	-0.04851	-0.1351
	p	0.3109	0.6222	0.3555	0.5271	0.6089	0.7626	0.7955	0.4688
	95% CI	-0.1457, 0.4287	-0.2712, 0.4324	-0.1592, 0.4173	-0.4117, 0.2214	-0.4051, 0.2476	-0.2813, 0.3744	-0.3961, 0.3113	-0.4671, 0.2303
IL-17	r	0.5033	0.4233	0.4880	0.2250	-0.4448	0.4919	0.3513	-0.5412
	p	**0.0005*****	0.0141	**0.0008*****	0.1685	**0.0058****	**0.0020****	**0.0487***	**0.0014***
	95% CI	0.07291, 0.5856	0.1382, 0.7000	0.05273, 0.5721	-0.09744, 0.5047	-0.672, -0.1411	0.1997, 0.7037	0.002837, 0.6236	-0.7487, -0.2372
TNF-α	r	0.06040	0.08432	-0.06521	-0.08602	0.01444	0.02736	0.07944	0.04398
	p	0.7225	0.6953	0.7014	0.6513	0.9407	0.8880	0.7121	0.8383
	95% CI	-0.2690, 0.3771	-0.3304, 0.4718	-0.3812, 0.2645	-0.4330, 0.2831	-0.3540, 0.3790	-0.3427, 0.3900	-0.3348, 0.4679	-0.3660, 0.4397
Cortisol	r	-0.1435	-0.09113	-0.09080	-0.04554	-0.07459	0.03552	0.3019	-0.2167
	p	0.3359	0.6140	0.5438	0.7802	0.6563	0.8416	0.0877	0.2259
	95% CI	-0.4137, 0.1499	-0.4213, 0.2604	-0.3684, 0.2017	-0.3521, 0.2699	-0.3852, 0.2512	-0.2893, 0.3495	-0.04629, 0.5847	-0.5213, 0.1369
Adiponectin	r	0.009301	-0.09972	0.01211	0.002145	0.008912	0.01358	0.2471	**0.1183**
	p	0.9505	0.5808	0.9356	0.9895	0.9577	0.9355	0.1657	0.5121
	95% CI	-0.2787, 0.2957	-0.4284, 0.2523	-0.2761, 0.2983	-0.3096, 0.3135	-0.3117, 0.3277	-0.3074, 0.3318	-0.1052, 0.5443	-0.2346, 0.4437
CCL1	r	-0.1102	-0.1341	-0.2122	-0.2997	0.03847	-0.09459	0.06505	**0.2083**
	p	0.4609	0.4567	0.1521	0.0603	0.8186	-0.4021, 0.2321	-0.2847, 0.3995	-0.1454, 0.5148
	95% CI	-0.3751, 0.2075	-0.4565, 0.2193	-0.4707, 0.07981	-0.5591, 0.01314	-0.2848, 0.3539	-0.4021, 0.2321	-0.2847, 0.3995	-0.1454, 0.5148
CCL2	r	-0.1509	-0.2443	-0.1143	-0.1332	0.2778	-0.2400	-0.2376	0.2881
	p	0.3464	0.1933	0.4653	0.4320	0.1061	0.1649	0.2062	0.1226
	95% CI	-0.4382, 0.1644	-0.5557, 0.1273	-0.4010, 0.1927	-0.4384, 0.1995	-0.06114, 0.5594	-0.5309, 0.1014	-0.5508, 0.1343	-0.08051, 0.5874

Table S4. Correlation of depressive scores and inflammatory biomarkers in female group

Inflammatory biomarkers		Indexes of depressive symptoms							
		BDI	STAI-X-1	HAMD-17	BIS	RSE	BHS	KSSI	RAS
**IL-1β**	**r**	0.3757	0.4714	0.3008	0.2235	-0.5629	0.4633	0.4718	-0.5568
	p	**0.0056****	**0.0019****	**0.0286***	0.1112	**<0.0001******	**0.0012****	**0.0018****	**0.0002*****
	95% CI	0.1173, 0.5865	0.1914, 0.6804	0.03313, 0.5282	-0.05265, 0.4679	-0.7334, -0.3258	0.1999, 0.6643	0.1920, 0.6807	-0.7381, -0.3006
**IL-6**	**r**	0.2365	0.2652	0.2578	0.2178	-0.3319	0.3270	0.2762	-0.3247
	p	0.0982	0.0981	0.0678	0.1287	**0.0297***	**0.0283***	0.0844	**0.0409***
	95% CI	0.03058, 0.5304	-0.05052, 0.5328	-0.01922, 0.4981	-0.06453, 0.4678	-0.5750, -0.03496	0.03694, 0.5662	-0.03867, 0.5412	-0.5778, -0.01462
**CRP**	**r**	0.2351	0.06551	0.2596	-0.1058	-0.06448	0.08959	-0.06686	0.09525
	p	0.0935	0.6880	0.0631	0.5271	0.6739	0.5584	0.6819	0.5588
	95% CI	-0.04051, 0.4774	-0.2512, 0.3696	-0.01441, 0.4973	-0.4117, 0.2214	-0.3514, 0.2335	-0.2095, 0.3734	-0.3707, 0.2499	-0.2229, 0.3951
**IL-17**	**r**	0.04092	0.1315	0.04571	0.01107	0.1279	-0.05875	-0.01130	-0.06777
	p	0.7711	0.2640	0.7452	0.9379	0.3970	0.6981	0.9441	0.6737
	95% CI	-0.2320, 0.3079	-0.2873, 0.3279	-0.2275, 0.3122	-0.2627, 0.2832	-0.1687, 0.4033	-0.3433, 0.2356	-0.3179, 0.2975	-0.3678, 0.2451
**TNF-α**	**r**	0.3043	0.2403	0.2486	0.1921	-0.2832	0.2935	0.2522	-0.2518
	p	**0.0446***	0.1710	0.1037	0.2172	0.0849	0.0737	0.1502	0.1508
	95% CI	0.008054 0.5514	-0.1066 0.5351	-0.05223 0.5080	-0.1150 0.4656	-0.5529 0.04023	-0.02896 0.5607	-0.09407 0.5440	-0.5437 0.09447
**Cortisol**	**r**	0.1718	0.05319	0.1304	0.2293	-0.07560	0.2395	0.04086	-0.02205
	p	0.2188	0.7412	0.3519	0.1021	0.6175	0.1089	0.7998	0.8912
	95% CI	-0.1034, 0.4225	-0.2588, 0.3551	-0.1450, 0.3871	-0.04662, 0.4726	-0.3581, 0.2196	-0.05469, 0.4954	-0.2703, 0.3443	-0.3275, 0.2876
**Adiponectin**	**r**	0.01797	-0.03279	0.05301	-0.1083	-0.05177	0.2395	0.1065	-0.01918
	p	0.8984	0.8387	0.7062	0.4448	0.7326	0.1089	0.5076	0.9052
	95% CI	-0.2536, 0.2869	-0.3371, 0.2777	-0.2205, 0.3188	-0.3703, 0.1697	-0.3371, 0.2422	-0.2254, 0.3528	-0.2081, 0.4011	-0.3250, 0.2903
**CCL1**	**r**	0.1018	0.1633	0.1012	0.2054	-0.1397	0.1615	0.2209	-0.3078
	p	0.4682	0.3075	0.4707	0.1441	0.3545	0.2837	0.1652	0.0503
	95% CI	-0.1733, 0.3622	-0.1520, 0.4485	-0.1739, 0.3617	-0.07160, 0.4530	-0.4133, 0.1571	-0.1352, 0.4316	-0.09320, 0.4949	-0.5623, -8.998
**CCL2**	**r**	0.1018	-0.1906	-0.03903	-0.1439	0.2238	-0.1348	-0.009729	0.1065
	p	0.4682	0.2450	0.7857	0.3187	0.1442	0.3831	0.9531	0.5187
	95% CI	-0.3436, 0.2049	-0.3113, 0.2392	-0.4061, 0.1401	-0.07835, 0.4883	-0.4151 0.1689	-0.4147, 0.2091	-0.3243, 0.3068	-0.2163, 0.4084

Supplementary figures

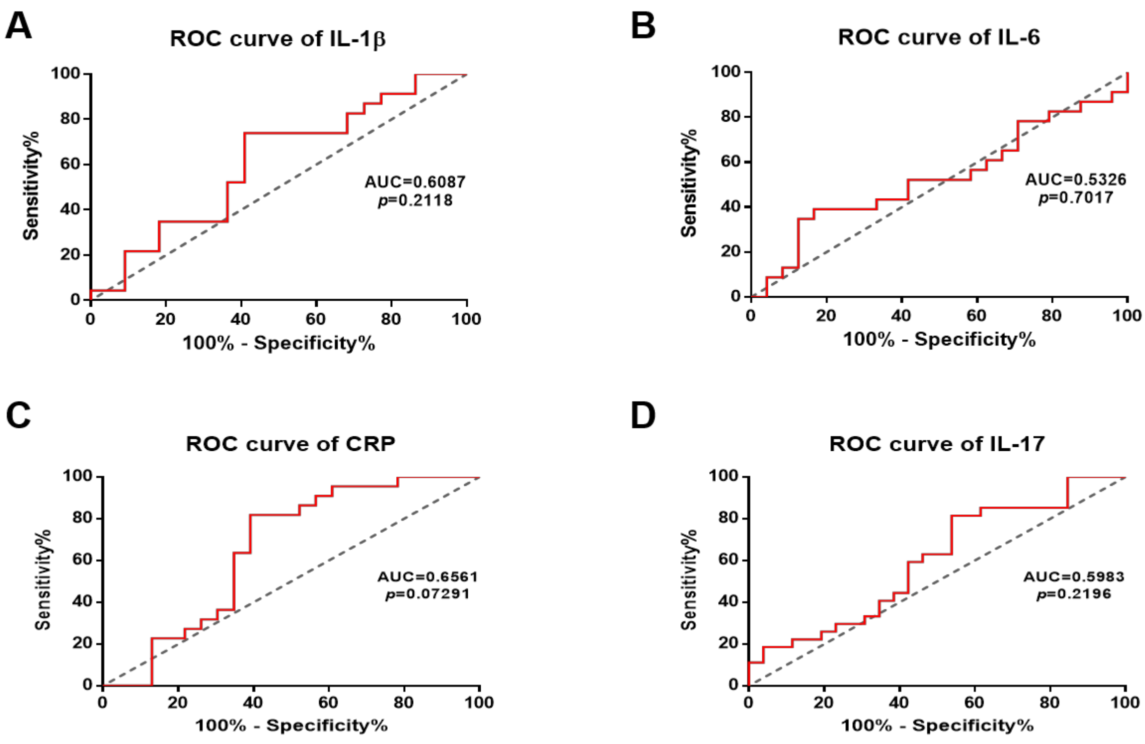

Fig. S1. The results of receiver operating characteristic (ROC) anaylysis for inflammatory biomarkers in young adulthood. (A-C) ROC curves of IL-1β, IL-6, and CRP in male group. (D) IL-17 in female group. Male (HC; n=24, MDD; n=23) and female (HC; n=26, MDD; n=27).
